# A Novel Panorama Depth Estimation Framework for Autonomous Driving Scenarios Based on a Vision Transformer

**DOI:** 10.3390/s24217013

**Published:** 2024-10-31

**Authors:** Yuqi Zhang, Liang Chu, Zixu Wang, He Tong, Jincheng Hu, Jihao Li

**Affiliations:** 1 College of Automotive Engineering, Jilin University, Changchun 130022, China; yuqi22@mails.jlu.edu.cn (Y.Z.); chuliang@jlu.edu.cn (L.C.); zixu23@mails.jlu.edu.cn (Z.W.); tonghe23@mails.jlu.edu.cn (H.T.); 2 National Key Laboratory of Automotive Chassis Integration and Bionics, Jilin University, Changchun 130022, China; 3 Department of Aeronautical and Automotive Engineering, Loughborough University, Loughborough LE11 3TU, UK; j.li12@lboro.ac.uk

**Keywords:** panorama depth estimation, autonomous driving, deep learning, feature fusion, intelligent vehicle

## Abstract

An accurate panorama depth estimation result is crucial to risk perception in autonomous driving practice. In this paper, an innovative framework is presented to address the challenges of imperfect observation and projection fusion in panorama depth estimation, enabling the accurate capture of distances from surrounding images in driving scenarios. First, the Patch Filling method is proposed to alleviate the imperfect observation of panoramic depth in autonomous driving scenarios, which constructs a panoramic depth map based on the sparse distance data provided by the 3D point cloud. Then, in order to tackle the distortion challenge faced by outdoor panoramic images, a method for image context learning, ViT-Fuse, is proposed and specifically designed for equirectangular panoramic views. The experimental results show that the proposed ViT-Fuse reduces the estimation error by 9.15% on average in driving scenarios compared with the basic method and exhibits more robust and smoother results on the edge details of the depth estimation maps.

## 1. Introduction

The safety of autonomous driving is contingent upon its perception ability to comprehensively understand its surroundings. It is essential to accomplish this that the autonomous driving systems must ascertain the relative distance between the vehicle and surrounding objects. This demand for high-quality omnidirectional ranging has prompted LiDARs to be widely used in autonomous driving. However, its high cost hinders the popularization of advanced autonomous driving technology and its sparse 3D scanning results can only provide limited scene-depth understanding. With the rapid development of computer vision, the depth estimation method for panoramic images in driving scenarios is expected to overcome the limitations of sparse LiDAR scanning results in scene understanding, leading to the realization of an affordable, high-density surround-type depth perception system.

Capturing scene depth from RGB images is a popular research topic of computer vision. Classical depth estimation methods obtain the scene depth based on the potential physical properties of images. The Multiple-View Stereo (MVS) algorithm uses the triangulation method to match two images and converts the disparity between them into depth [1]. The Structure from Motion (SFM) algorithm mainly utilizes the matrix information to convert the motion parameters and estimates the depth of the feature points [2]. Moreover, monocular depth estimation methods generally recovered depth from defocus (DFD) in RGB images based on optical principles in earlier studies [3]. The ground-breaking advances in deep learning in computer-vision tasks have significantly enhanced the performance of depth estimation [4,5]. The pre-trained neural network has been used for end-to-end dense depth estimation from a single image, and the innovations in network structures, loss functions and training strategies of deep learning have outstandingly improved the estimation accuracy.

The large-scale application of the Around View Monitor (AVM) in vehicles has further enhanced the application potential of depth estimation in autonomous driving. The AVM relies on a set of surrounding cameras to capture background images in different directions and constructs a panoramic image based on the current vehicle position through an image synthesis algorithm. Depth estimation on the panoramic images can break through the limited view of traditional monocular cameras and estimates the distance from the center camera to the surrounding environment with a 360° fusion perspective. Panorama depth estimation thus is critical to comprehend the scene information of the real 3D world. It has a strong potential to be utilized in panorama parking systems, panorama reversing systems, traffic jam assistance systems, and 3D scene reconstruction.

The panoramic image offers a comprehensive view of the scene based on their multi-view and wide-format characteristics. However, these characteristics also lead to distortion problems when such images are represented in the equirectangular projection (ERP). During the camera scanning and imaging process, the distortion of panoramic images increases from the center to the sides along the latitude direction, and the object distance that increases along with the scanning angle leads the scale of the image to shrink gradually from the center to both sides. These phenomenon result in the deformation of objects within the image, damaging the semantic information of the image seriously, which lead to biased edge depth estimation and catastrophic estimation errors.

Imperfect observation, which is difficult to comprehensively obtain complex environment information with the limitation on algorithm and equipment, also become a major challenge, hindering the application of panorama depth estimation in autonomous driving. To support the progression of the panorama depth solutions, several datasets have been produced. Current outdoor panoramic image datasets lack comprehensive coverage of road traffic scenes, making it challenging to fully address various driving conditions. As a result, advanced panorama depth estimation solutions are often developed and validated using indoor scene datasets, such as Matterport3D [6], Stanford2D3D [7], PonoSUNCG [8], and 360D [9].

The current methods of panoramic image acquisition face limitations in providing consistent and accurate depth information, especially in complex driving environments. These issues include inconsistent scene-depth understanding due to sparse or incomplete data. Additionally, existing panoramic depth estimation approaches struggle to effectively capture detailed depth information, limiting their reliability in real-world driving scenarios. To address these challenges, we propose an end-to-end panoramic depth estimation framework, which improves depth estimation performance through innovations in data augmentation and neural network structure.

The proposed framework includes a Patch Filling module, which employs mean interpolation on 3D point clouds to generate dense depth maps, effectively compensating for missing data. Additionally, we introduce the ViT-Fuse model, which leverages the spatial encoding power of a Vision Transformer [10] to optimize feature fusion and mitigate edge distortion in panoramic depth estimation. Together, these components enable more accurate and reliable depth perception, advancing the capability of autonomous vehicles to navigate complex environments with greater precision. The main contributions of this study are as follows:

In this study, we propose a novel framework for panoramic depth estimation that makes several key contributions to the field of autonomous driving. First, we introduce a Patch Filling module designed to address the sparsity inherent in 3D point cloud data by generating dense depth maps. These panoramic depth maps, integrated into the Ford Campus Vision and LiDAR Dataset [11], enhance the dataset’s usability for future research and demonstrate improved depth estimation accuracy in autonomous driving scenarios through comparative experiments. Additionally, we develop a new panoramic projection fusion model, ViT-Fuse, which leverages the robust spatial encoding capabilities of a Vision Transformer to mitigate the distortion challenges associated with equirectangular projection (ERP) images. This model outperforms conventional methods, particularly in outdoor environments, providing smoother and more accurate edge details in the depth maps. Our experimental results further highlight the practical potential of this framework, achieving a maximum error reduction of 19.36% compared to baseline models and demonstrating superior accuracy, especially under the tightest threshold conditions. These findings establish ViT-Fuse as an excellent solution for enhancing panoramic depth estimation in complex driving environments.

The remainder of the paper is structured as follows: Section 2 reviews related works on the autonomous driving panorama depth perception schemes. In Section 3, the Patch Filling module and the processing method for autonomous driving datasets are presented. Section 4 detailed expounds the basic structure and inference principles of the ViT-Fuse model. In Section 5, the experimental settings and the analysis and comparison of the experimental data are shown. We discuss the experimental results in Section 6. Lastly, in Section 7, the conclusions are provided.

## 2. Related Work

### 2.1. Autonomous Driving Panorama Perception Schemes

KITTI [12] is a standard test dataset for autonomous driving. It uses two color cameras, two grayscale cameras, a Velodyne 3D laser scanner, and other devices to collect data. The stereo information of the dataset mainly collected by the binocular cameras, which used to restore the 3D scene structure from different angles. This helps sense the vehicle’s surrounding environment and obtain 3D depth information. WoodScape [13] is the first autonomous driving dataset released by Valeo based on fisheye cameras. This dataset can be used to achieve a panoramic view through fisheye cameras distributed in four directions on the vehicle. Although fisheye cameras can provide a wide field of view, the projected geometry it presents is much more complex, which means that the image displayed by the fisheye camera is distorted. The distortion of the fisheye camera images is severe, and correcting these images may lead to losing part of the field of view. Therefore, de-distortion methods and vision algorithms are highly desired in the panorama perception scheme of WoodScape. The Málaga Stereo and Urban [14] dataset comprises a sequence of images recorded in different parts of the city; it covers various situations in the city and provides data such as stereo images captured by stereo cameras, IMU, GPS ground truth trajectories, and 3D point clouds obtained by multiple laser scanners in different directions. In addition, it also demonstrates the 3D reconstruction of driving scenarios through GPS point interpolation. ApolloScape [15] collects point clouds from Reigl using mobile LiDAR scanning instruments. The 3D point cloud produced by this method is usually more accurate and denser. This dataset uses a calibrated high-resolution camera installed on the roof of a car to simultaneously record and capture the scenes around the car at a rate of one frame per meter. These scenes can be used to describe a high-grained static 3D world.

In comparison to other computer-vision tasks, the availability of panoramic datasets designed specifically for autonomous driving is limited. These datasets can only provide sparse point cloud data as an imperfect observation of the distance of surrounding objects, resulting in a reduced generalization of prediction schemes. Therefore, they are not directly suitable for panorama depth prediction. As a result, it is imperative to carefully execute appropriate data processing techniques to provide accurate ground truth for the development of panorama depth prediction.

### 2.2. Depth Estimation Based on RGB Images

Depth estimation based on single or panoramic RGB images has become the mainstream research with its advantages of lost cost and richer captured perception information. Make3D [16] and KITTI are real-scene datasets commonly used in monocular depth estimation tasks, while Matterport3D and Stanford2D3D are panoramic datasets commonly used in panorama depth estimation tasks. The traditional application of machine learning methods in monocular depth estimation uses a Markov Random Field (MRF) to construct a probabilistic graphical model of the depth relationship. However, this method is difficult to apply to natural scenes due to its complexity and low estimation accuracy. In contrast, deep learning methods have shown promising results in monocular depth estimation. Eigen et al. [17] first applied the deep neural network to the monocular depth estimation. They proposed the coarse-scale network to predict the picture’s global depth and the fine-scale network to optimize the local details. In addition, the convolutional neural network (CNN) extract image features and abstract them layer by layer through the deep neural network, which is used to complete the depth estimation tasks for high-dimensional RGB images. Liu et al. [18] combined deep convolutional neural networks with continuous conditional random fields for deep estimation. Laina et al. [19] proposed a fully convolutional network based on residual learning.

In order to break through the field of view limitation, panoramic RGB images are fused with multi-view scene information with the cost of severe distortion. Variations in image characteristics have changed the method of depth estimation. Some studies in the depth estimation of panoramas with an improved convolutional method adapt to the panoramic image. For example, Tateno et al. [20] designed distortion-aware convolutional filters to expand the receptive field. Fernandez et al. [21] proposed the EquiConv that applies deformable convolutions to accommodate spherical geometry. Chen et al. [22] utilized strip pooling and deformable convolution to build an encoding structure that can adapt to different degrees of panoramic distortion. As many depth estimation methods based on RGB images mainly adopt supervised regression models, which require the RGB images to have depth label information, unsupervised estimation methods thus have gradually become the leading research direction without the requirement of depth label information considering the poor performance and high cost of original depth information. For example, Garg et al. [23] proposed exploiting stereo images, and Godard et al. [24] exploited the consistency of left and correct views for unsupervised depth estimation.

Newer studies have also explored various approaches to better capture both local and global information. Jin et al. [25] emphasized the role of geometric structures for indoor depth estimation. Bhat et al. [26] adaptively split depth ranges into bins, improving accuracy across diverse scenes. Bhat et al. and Dosovitskiy et al. [10] demonstrated that transformer-based methods outperform CNNs in depth estimation for images on large-scale datasets. PCFormer [27] merges convolutional and transformer architectures in parallel to balance local and global feature extraction in 360° depth estimation. Additionally, MCPDepth [28] applies stereo matching across cylindrical panoramas, achieving a robust performance on large outdoor datasets like Deep360. Peng et al. [29] proposed a high-resolution framework for depth estimation that aligns perspective and panoramic depth maps, producing finer predictions with fewer artifacts, especially on high-resolution datasets. These advancements reflect a shift toward more flexible and adaptive models capable of handling the unique challenges of panoramic RGB images.

### 2.3. Distortion Mitigation for Panorama Depth Estimation

The main challenge of depth estimation for panoramic images is the adverse effect of image distortion on the estimation performance. Due to technical limitations, traditional depth estimation methods are no longer able to achieve satisfactory results when it comes to addressing the distortion problem in panoramic images. Besides enhancing convolution techniques, several studies have focused on exploring alternative approaches to mitigate distortion, including fusion methods utilizing the equirectangular projection and cubemap projection (CMP). Unlike ERP images that provide comprehensive image information for panoramic representation but suffer from severe distortion, CMP images can be presented without distortion. However, CMP images exhibit information discontinuities on the edges of the cube structure. Therefore, Wang et al. [30] proposed the BiFuse, which uses dual projections to fuse and complement equirectangular features and cubemap features, improving the fault tolerance of the model to distortion. UniFuse [31] discards a decoder that predicts cubemap features based on BiFuse, reducing the complexity of the model. Shen et al. [32] proposed a fusion method using 45°-rotated double cubes, which can also reduce the influence of distortion. However, this method needs to repeatedly change the projection for fusion, which undoubtedly increases the overall complexity of the model. In addition, the less distortion property of tangent images has also received attention. For example, Eder et al. [33] used a set of tangent images to represent panoramas, which can effectively utilize CNN. However, they ignored the unavoidable differences between the tangent images decomposed from the panorama. This gap makes the estimated depth of the same object in different tangent images inconsistent, which reduces accuracy in the final merging. SliceNet [34] leverages vertical slices to maintain geometric consistency in depth estimation, achieving high accuracy on datasets. However, the slicing approach overlooks the distortion characteristics along latitudinal lines, making it challenging to accurately estimate depth in regions close to the poles.

In response to these challenges, newer methods have been proposed. Shen et al. [35] utilizes a transformer architecture with spherical-domain token flows, mitigating distortions while enhancing the model’s ability to perceive geometric structures. Zhuang et al. [36] applies self-supervised learning with edge-aware loss functions, leveraging spherical geometry to reduce artifacts and improve depth predictions at object boundaries. Bai et al. [37] introduces a global-to-local strategy using a Cubemap Vision Transformer (CViT), which captures both global and local features effectively to handle panoramic distortions. Further advancements include AFNet [38], which employs asymmetric fusion between ERP and CMP projections to guide feature extraction, resulting in enhanced performance across multiple panoramic datasets.

## 3. Patch Filling

**Figure 1:**
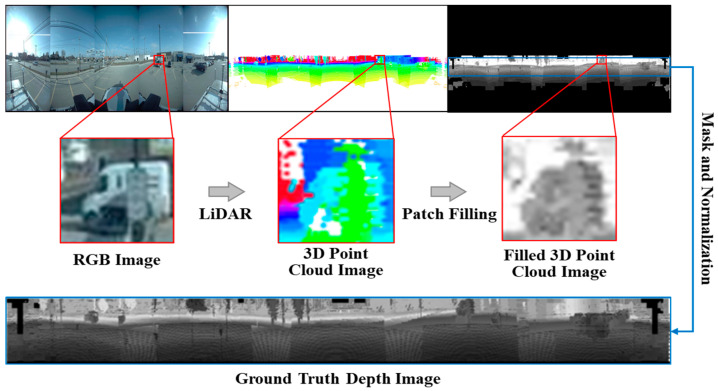
The three images are represented from top to bottom as an RGB image, point cloud projection map, and processed true depth map.

We utilized the Ford Campus Vision and LiDAR Dataset [11] for our outdoor autonomous driving scene dataset. This dataset provides panoramic images with comprehensive scene information, created by stitching together images from a five-camera omnidirectional camera system, and raw 3D point cloud data are provided via laser scanner. However, the Ford Campus Vision and LiDAR Dataset does not directly offer panoramic depth maps. Therefore, in this section, we generate panoramic depth maps using 3D point cloud data and match them to their corresponding panoramas, with the aim of using these panoramic depth maps as the ground truth for subsequent analysis. Additionally, the generated panoramic depth maps are integrated into the dataset, enabling other researchers to utilize them directly. We project the raw 3D point cloud data onto a blank image that has the same size as the corresponding RGB image in the dataset. The points in the projection image are colored based on their distances. However, the point cloud data need to be denser or even be supplemented in certain areas. As shown in Figure 1, there are some blank areas in the corresponding 3D point cloud image of the scene information, such as vehicles in the framed area of the RGB image, which indicates that the rendered point cloud data are not complete. The insufficient availability of 3D point cloud data from critical surrounding objects can significantly impede the integrity of the ground truth for depth estimating. This presents an undeniable challenge to the subsequent development of the panorama depth estimation.

In this paper, we propose the Patch Filling method, which utilizes a fixed-size square slider to fill the points within the range of point cloud projection. The filling conditions and process are determined by the information of the original points already present in the slider. The blank areas in the projection correspond to the positions where the distance information from point cloud data is missing. We can identify and fill the missing information in the matrix corresponding to the blank pixels using the Patch Filling method, by converting the pixels of the ERP image into a two-dimensional matrix. The specific filling formula is as follows:

$$C_{w}=\frac{\sum_{i=1}^{r^{2}}C_{i}-255\times N_{w}}{r^{2}-N_{w}}$$

where *r* is the number of rows or columns of the square slider matrix and *C_i_* is the matrix element value corresponding to the *i*th pixel in the slider area. *C_w_* is the matrix element value corresponding to the blank pixel in the slider area after being filled. *N_w_* is the number of blank pixels in the slider area.

The slider size and the filling conditions were selected by grid search, the termination condition of the search are that the slider size must end at the given max value and the blank pixels need to be less than 70% of all pixels. It ensures the feasibility of the Patch Filling method and the quality of the generated depth map after filling. The result of filling the framed area is shown in Figure 1. After applying the Patch Filling method (Algorithm 1), the missing distance information from the point cloud data of the vehicle is supplemented, which is nearly consistent with the actual situation. The image obtained after accomplishing the slider filling demonstrates that the sparseness problem has been solved. However, the driving scenarios generally do not pay attention to the information near the poles that is far away from the vehicle in the panorama, considering the needs for research in autonomous driving. Therefore, we employ masking in the area outside the range of 3D point cloud projection and normalize the remaining areas. The ground-truth image, which is shown in Figure 1, represents the expected depth map corresponding to the panorama. Then, the panoramic image will combine with the ground-truth image within the panorama depth estimation network to carry out subsequent estimation tasks.
**Algorithm 1** The Patch Filling algorithm for the proposed method**Input:** sparse depth map **Output:** dense depth map 1:    **for** r = 5, 10, 20 **do** 2:       **for** m from 583 to 998 step r 3:          **for** n from 0 to 2400 step r 4:             B ← rgb[m:m + r, n:n + r] 5:             C ← B.reshape(−1) 6:             num ← 0 7:             **for** i in C **do** 8:                  **if** i = 255 **then** 9:                   num ← num + 1 10:                **end if** 11:            **end for** 12:            su ← $\sum_{i=1}^{r^{2}}C_{i}$ 13:            s ← 0 14:            **if** num < 0.7r^2^ **then** 15:               **for** i in C **do** 16:                  **if** i = 255 **then** 17:                      C[s] ← (su − 255num)/(r^2^ − num) 18:                  **end if** 19:                  s ← s + 1 20:               **end for** 21:            **end if** 22:            D ← C.reshape(B.shape) 23:            rgb[m:m + r, n:n + r] ← D 24:         **end for** 25:      **end for** 26:   **end for** 27:  **return** rgb

## 4. Panorama Depth Estimation Network

The continuous development of deep learning has endowed panorama depth estimation with a major breakthrough in feature fusion and the self-learning of panoramic images. We proposed a panoramic feature fusion model, ViT-Fuse, improving the method of extracting equirectangular image features in UniFuse and integrating the ViT module in the encoding stage to realize image context learning for equirectangular panoramas, so that it can obtain more comprehensive equirectangular features, enhancing the fusion features and improving the accuracy of depth estimation. The subsequent subsections explain in detail the structure and inference principles of the ViT-Fuse model.

### 4.1. Panorama Projection Fusion

**Figure 2:**
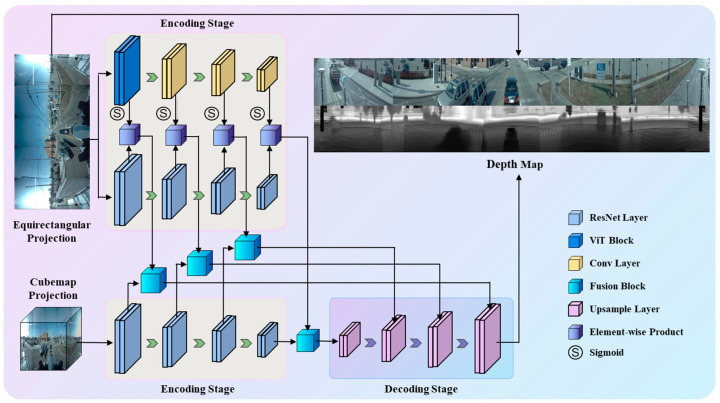
The architecture of the proposed ViT-Fuse.

The proposed ViT-Fuse model demonstrates a new panorama projection fusion scheme. The overall structure ViT-Fuse is shown in Figure 2; it uses the UniFuse network [31] as the backbone. ViT-Fuse primarily comprises the pre-trained ResNet-18 [39] and performs the fusion process from the CMP branch to the ERP branch only at the decoding stage. The ERP image is typically chosen as the representative format for panoramic views and depth maps due to the limited field of view and discontinuous scenes captured by CMP. Therefore, similar to UniFuse, ViT-Fuse reduces a decoder for predicting the cube depth maps, which may prevent the training from losing focus on the equirectangular depth. In the ViT-Fuse structure, two encoders are used to extract feature maps from the equirectangular image and the cubemap. Moreover, the ERP branch obtains new ViT features utilizing the additional ViT module, which can be fused with equirectangular features generated by the ResNet-18 network to supplement the feature information. The enhanced equirectangular features and cubemap features pass through the fusion module in the model to improve the result of the feature fusion and obtain more accurate depth estimation.

### 4.2. Vision Transformer Module

Previous studies have focused on conducting experiments on indoor scene datasets, such as Stanford2D3D, Matterport3D, and 360D. For outdoor-scene-related tasks, such as autonomous driving, panoramic images encompassing a wider range of targets tend to exhibit greater comprehensiveness. However, problems may arise while attempting to capture critical objects displayed within smaller ranges in the image, which can potentially ignore the edge features of said objects and result in the challenging panorama depth estimation.

Research on ViT techniques have established the practicability and advantages of splitting an image into multiple patches of identical sizes and leveraging transformer encoders to process them. As each patch undergoes processing, positional information is embedded to enable its integration into the transformer encoder, which consists of multiheaded self-attention (MSA) and Multi-Layer Perceptron (MLP) blocks. ViT facilitates the learning of global image features, thereby aiding in capturing detailed panorama information.

In our framework, the ViT module works in conjunction with ResNet-18 to enhance depth estimation performance. ResNet-18 extracts initial feature maps from both ERP and CMP images, which are refined through its layers to capture local details. Simultaneously, ERP images are also fed into the ViT module, which splits the image into patches, flattens them, and encodes positional information for each patch. The resulting ViT-encoded features are further processed through convolutional layers to produce normalized equirectangular features. These features are crucial for maintaining accuracy in object edges and small-scale details that might otherwise be lost.

The integration of ViT with ResNet enhances the depth estimation model by combining ResNet’s local feature extraction capabilities with ViT’s strength in capturing global representations, leading to improved predictive performance. ResNet preserves essential local features, while ViT addresses the limited receptive field of convolutional layers by providing global context, enhancing the model’s robustness in diverse autonomous driving environments. This fusion helps the model mitigate distortions inherent in panoramic images and improves its ability to capture fine-grained details at object edges. Specifically, the self-attention mechanism of ViT further enables the model to learn global dependencies, overcoming CNNs’ limitations in handling long-range relationships. This leads to more precise depth predictions, especially in complex geometrical scenes.

In light of depth prediction tasks and model frameworks, alterations have been made to the original ViT, including the elimination of the classification head while forgoing the use of learnable embedding to the sequence of embedded patches. In an effort to facilitate the subsequent feature fusion, the ViT output is preprocessed and used as the initial ViT feature. The formulation is expressed as follows:

$$z_{0}=\left[x_{p}^{1}E;x_{p}^{2}E;\ldots ;x_{p}^{N}E\right]+E_{pos},\;\;E\in R^{\left(P^{2}\cdot C\right)\times D},E_{pos}\in R^{N\times D}$$

$$z_{k}^{\prime }=MSA\left(LN\left(z_{k-1}\right)\right)+z_{k-1},\;\;k=1\ldots L$$

$$z_{k}=MLP\left(LN\left(z_{k}^{\prime }\right)\right)+z_{k}^{\prime },\;\;k=1\ldots L$$

$$\left[y_{1};y_{2};\ldots ;y_{N}\right]=LN\left(z_{L}^{n}\right),\;\;n=1\ldots N$$

$$F_{vit}^{0}=T\left(\left[y_{1};y_{2};\ldots ;y_{N}\right]\right)$$

where *E* represents the fully connected layer, *E_pos_* represents the position embeddings, *x_p_* is the sequence of flattened 2D patches, *P^2^* is the resolution of each image patch, *D* represents the size of the constant latent vector used by transformer through all of its layers, *LN* means Layernorm, and *T* (*·*) means preprocessing.

### 4.3. Encoding Stage

The ViT-Fuse model retains ResNet-18, wherein the ERP image and its corresponding CMP image are fed to the equirectangular encoder and cube encoder located within ResNet-18, respectively, and the corresponding initial features are obtained first. Subsequently, the initial features undergo a process of max-pooling before being channeled through the first ResNet layer, leading to the acquisition of the corresponding equirectangular and cubemap features. The formulation is expressed as follows:

$$F_{equi}^{1}=R_{L}^{1}\left(M\left(F_{equi}^{0}\right)\right)$$

$$F_{equi}^{a}=R_{L}^{a}\left(F_{equi}^{a-1}\right),\;\;a=2,3,4$$

$$F_{cube}^{1}=R_{L}^{1}\left(M\left(F_{cube}^{0}\right)\right)$$

$$F_{cube}^{a}=R_{L}^{a}\left(F_{cube}^{a-1}\right),\;\;a=2,3,4$$

where *M* (·) represents the max-pooling of the feature map, *R_L_* (·) represents the ResNet layers, and *F_equi_* and *F_cube_* represent the equirectangular features and cubemap features output through the ResNet layers, respectively. Simultaneously, the ERP images also pass through the previously mentioned ViT module. Analogous to the ResNet layers in the equirectangular encoder and cube encoder, the initial equirectangular features obtained by ViT pass through convolutional layers and directly yield the related features. These features first generate the normalized equirectangular features *F_vit_* through the application of the sigmoid function. *F_vit_* is multiplied with *F_equi_* to obtain the equirectangular features *F_enc_* with more comprehensive features after undergoing feature enhancement. Afterwards, *F_enc_* collaborates with *F_cube_* to complete the fusion of the two branch image features through the fusion module and ultimately obtain the fused features. Our fusion module still utilizes the CEE module in UniFuse as the fusion layer. The formulation for the above process is expressed as follows:

$$F_{vit}^{b}=S\left(Conv_{2}\left(F_{vit}^{b-1}\right)\right),\;\;b=1,2,3,4$$

$$F_{enc}^{c}=F_{equi}^{c}⊗F_{vit}^{c},\;\;c=0,1,2,3,4$$

where *Conv*_2_ denotes convolution with a kernel size of two, *S* (·) denotes the sigmoid function, and ⊗ denotes elementwise multiplication.

### 4.4. The Fusion Module

**Figure 3:**
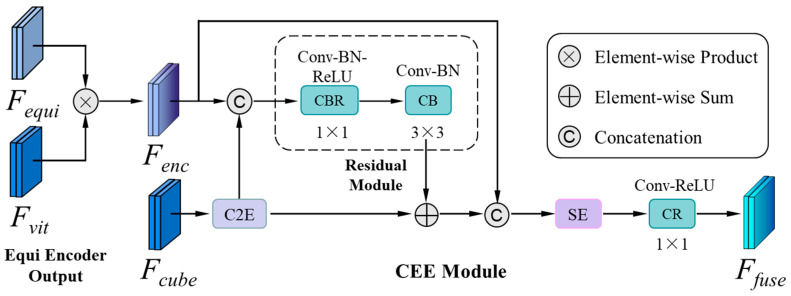
The architecture of the fusion module.

The structure of the fusion module we used is shown in Figure 3. Prior to the fusion with *F_enc_*, *F_cube_* undergoes a conversion process via the C2E module, followed by reprojection of the resulting features onto an equirectangular grid. They are then input into the CEE module to merge the two features. The corresponding formulation is expressed as:

$$F_{fuse}=CEE\left(F_{enc},C2E\left(F_{cube}\right)\right)$$

where *F_fuse_* denotes the fused features, *C*2*E* (*·*) represents the reprojection of the cubemap features in the equirectangular images, and *CEE* (*·*) denotes the process contained in the CEE module in the structure. *CEE* is one of the modules in the fusion framework of ViT-Fuse designed by Jiang et al., which use cubemap to enhance the equirectangular features. A residual module is included in *CEE* to alleviate the hindrance caused by the discontinuity of CMP, which consists of a 1 × 1 conv and a 3 × 3 conv. In the fusion process, the enhanced equirectangular features and the *C*2*E* features output by the *C*2*E* module are fed to the *CEE* module first. Then, the two are concatenated, and the concatenated features are passed through the residual module, followed by two convolution modules. The 1 × 1 conv in the residual module can reduce the number of channels doubled due to concatenation, and the residual features are produced by passing through the 3 × 3 conv. The residual features are added to the *C*2*E* features. After the residual modulation, the missing features on the edge of images due to the discontinuity of the CMP can be filled. After concatenation of the enhanced *C*2*E* features and equirectangular features, a Squeeze-and-Excitation (*SE*) block is added in CEE before the final 1 × 1 conv recalibrates channel-wise feature responses to achieve better feature fusion. The process in *CEE* can be expressed as follows:

$$CEE\left(F,F^{\prime }\right)=CR\left(SE\left(Concat\left(F,F^{\prime }⊕CB\left(CBR\left(Concat\left(F,F^{\prime }\right)\right)\right)\right)\right)\right)$$

CBRF=ReluBnConv1FCBF=BnConv3FCRF=ReluConv1F

where Re*lu* (*·*) represents the Relu activation function, *Concat* represents the cross-channel concatenation, *Bn* represents BatchNorm, and ⊕ represents elementwise addition.

### 4.5. Decoding Stage

During the decoding stage, each upsampling layer corresponds to a respective layer at the encoding stage. The fused features obtained by passing through the layers at the encoding stage can be provided to the corresponding upsampling layers. The output of the last layer is directly fed into the first upsampling layer, while the feature of each remaining layer is concatenated with the output of the previous upsampling layer before passing through its corresponding upsampling layer. ERP images are progressively enlarged layer by layer throughout the process until the output of the last upsampling layer is obtained. Finally, the depth map predicted by the entire model is generated upon processing this output. The formulation for the above process is expressed as follows:

$$X^{d+1}=\left\{\begin{array}{l} Up_{\times 2}\left(Elu\left(Conv_{3}\left(F_{fuse}^{d}\right)\right)\right),\;d=4\\ UP_{\times 2}\left(Elu\left(Conv_{3}\left(Elu\left(Conv_{3}\left(Concat\left(X^{d+2},F_{fuse}^{d+2}\right)\right)\right)\right)\right)\right)\\ Elu\left(Conv_{3}\left(X^{d+2}\right)\right),\;\;d=-1 \end{array}\right.,\;d=3,2,1,0$$

$$X_{depth}=Conv_{3}\left(X^{0}\right)$$

$$P_{depth}=M_{depth}\cdot S\left(X_{depth}\right)$$

where *Up*_×2_ denotes upsampling by a factor of two, *Elu* (*·*) denotes the Elu activation function, *X* denotes the ERP image output by each layer, *X_depth_* denotes the depth prediction map before processing, *M_depth_* denotes the maximum depth, and *P_depth_* denotes the depth prediction map of the final output of the model.

### 4.6. Evaluation Metrics and Loss Function

The quantitative evaluation metrics we used to evaluate depth estimation include the Mean Absolute Error (MAE), Absolute Relative Error (AbsRel), Root Mean Squared Error (RMSE), and accuracy metric; the corresponding formulation is expressed as follows:

MAE=1m∑i=1myi−y^i,AbsRel=1m∑i=1myi−y^iyi,RMSE=1m∑i=1myi−y^i2,maxy^iyi,yiy^i<Thr

where *m* represents the total number of pixels, *y_i_* represents the actual depth value corresponding to the *i*th pixel, ${\hat{y}}_{i}$ represents the estimated value corresponding to the *i*th pixel, and *Thr* is the threshold. The accuracy metric usually uses three different thresholds, 1.25, 1.25^2^, and 1.25^3^. Theoretically, the higher the accuracy is, the better the effect of prediction is. In addition, this study still uses BerHu loss as the regression function in the model, and its formulation is expressed as follows:

$$\beta_{\delta }\left(g,p\right)=\left\{\begin{array}{ll} \left|g-p\right| & for\left|g-p\right|\leq \delta \\ \frac{\left|{\left(g-p\right)}^{2}\right|+\delta^{2}}{2\delta } & otherwise \end{array}\right.$$

## 5. Experiments and Results

We validated the effectiveness of the Patch Filling method in addressing the issue of sparse point clouds and improving depth estimation by comparing the ViT-Fuse model with and without Patch Filling. Additionally, we evaluated the proposed ViT-Fuse and compared it with UniFuse by conducting experiments on the Ford Campus Vision and LiDAR Dataset.

### 5.1. Datasets

The Ford Campus Vision and LiDAR Dataset is a real-world driving scenario dataset collected by an autonomous ground vehicle testbed. The test vehicle is equipped with a Velodyne 3D LiDAR scanner, push-broom forward-looking Riegl LiDARs, a Point Gray Ladybug3 omnidirectional camera system, and so on. Unlike datasets such as Matterport3D, this dataset does not directly provide raw depth maps but provides files such as 3D point cloud data. Ford Campus Vision and LiDAR Dataset pay more attention to the partial panoramic area mainly based on the driving perspective, and the point cloud data is also mainly distributed in this area. The dataset contains 3817 panoramic RGB images, of which 3800 are used as the training set for the experiment. During data processing, the corresponding panoramic actual depth maps are converted from the original 3D point cloud data.

### 5.2. Implementation Details

We implemented our experiments using PyTorch and trained on GEFORCE RTX 3090Ti GPU. We used Adam with default parameters as the optimizer with a constant learning rate of 1 × 10^−4^. During training, we set the input size to 512 × 1024 and the batch size to 1. At the encoding stage of the equirectangular images, the patch size in the ViT module we added was set to 8 × 8, the embedding dim was set to 1024, and the depth of the transformer encoder was 4.

### 5.3. Effectiveness of Patch Filling

We compared the effectiveness of Patch Filling (PF) in improving depth estimation by evaluating the ViT-Fuse model with and without PF on the Ford Campus Vision and LiDAR Dataset. The PF method was introduced to fill the gaps in sparse point cloud data, aiming to generate a denser and more accurate depth map. Without PF, the model suffered from incomplete depth information, particularly in regions where the point cloud data were sparse or missing. By applying PF, we can enhance the completeness of the depth map, leading to improved accuracy, especially in challenging areas such as object edges and distant objects.

**Figure 4:**
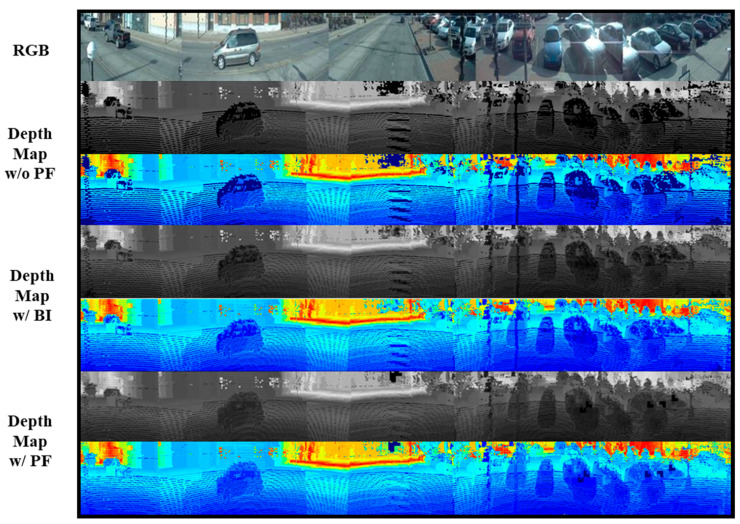
The visualization of the effectiveness of Patch Filling.

Figure 4 shows a visualization of the effectiveness of the PF method. The PF method effectively reduced the gaps caused by sparse 3D point clouds, generating denser panoramic depth maps that more accurately reflect real-world scenarios. Depth prediction results obtained using ViT-Fuse with depth maps supplemented by the PF method showed clear improvements. By utilizing these enhanced depth maps, the model benefitted from more reliable input, leading to more accurate predictions and a significant reduction in the adverse effects caused by sparse or incomplete observations. This demonstrated the effectiveness of the PF method in improving the overall depth estimation process for complex driving environments.

Table 1 demonstrates the quantitative results of our experiments. The ViT-Fuse model with PF achieved a significant reduction in error metrics compared to the version without PF. Specifically, the Mean Absolute Error (MAE) was reduced by 14.49%, the Absolute Relative Error (Abs Rel) was reduced by 18.03%, and the Root Mean Squared Error (RMSE) was reduced by 11.93%. These improvements indicated that PF effectively mitigated the gaps caused by sparse point cloud data, resulting in more accurate depth predictions. Furthermore, the accuracy metrics, particularly δ < 1.25, also showed substantial improvements, with an increase of 9.73%. This improvement highlighted the model’s ability to better capture fine details in the depth map, leading to more reliable predictions in autonomous driving scenarios. The results demonstrated that the inclusion of PF not only enhanced the overall accuracy of the depth estimation but also improved the model’s robustness in complex driving environments.

To further highlight the advantages of PF, we also conducted a comparative evaluation with Bilinear Interpolation (BI), a commonly used method for filling missing data in images [40]. BI excels in interpolation strength and is computationally efficient, which makes it suitable for simpler tasks. However, our experiments on the Ford Campus Vision and LiDAR Dataset, a complex outdoor dataset, revealed certain limitations of BI. Although BI can effectively fill sparse points, the interpolated depth maps it generates are often not smooth enough and lack the natural continuity found in real-world depth data. This discrepancy is especially evident in complex scenes, where the interpolation across the same object results in inconsistent depth values between neighboring regions. As a result, BI’s interpolation may appear fragmented, introducing artifacts that reduce the overall prediction quality. In contrast, PF addresses these issues by maintaining a smoother and more continuous depth map, as shown in Figure 4. PF not only fills missing data points but also preserves consistency across the depth values of objects, leading to more realistic depth predictions. This makes PF more effective in autonomous driving environments, where consistent depth estimation is crucial for tasks like object detection and obstacle avoidance.

The quantitative results in Table 1 further validate the advantages of PF over BI. PF achieved a reduction of 6.83% in MAE, 8.22% in Abs Rel, and 4.72% in RMSE, demonstrating improved overall performance in depth estimation. Additionally, the accuracy metric δ < 1.25 showed a 3.49% increase, indicating better prediction reliability. These results confirmed that PF offered a more effective solution for addressing the sparsity of point cloud data compared to BI, enhancing the performance and robustness of the ViT-Fuse model in real-world autonomous driving scenarios.

### 5.4. Comparative Results Between ViT-Fuse and Baseline Models

**Figure 5:**
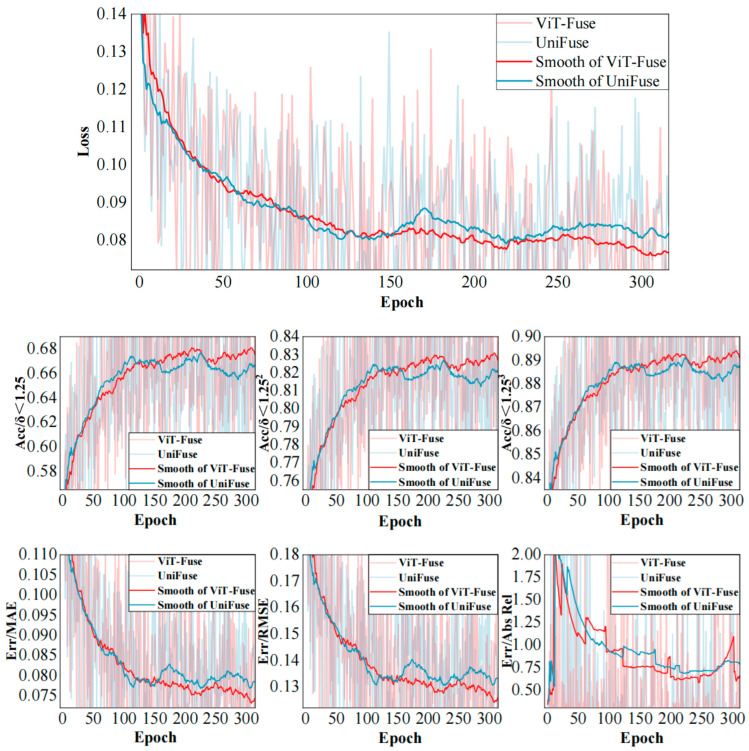
Curve comparison between ViT-Fuse and UniFuse.

The quantitative comparison between our model and several baseline models, including FCRN [19], BiFuse, and UniFuse, on the Ford Campus Vision and LiDAR Dataset is displayed in Table 2. As shown in Figure 5, the loss curves of ViT-Fuse and UniFuse exhibited little variance in the first 150 epochs, with UniFuse showing slightly better training efficacy. However, after 150 epochs, ViT-Fuse ultimately outperformed UniFuse across all metrics and maintained its lead. The inclusion of FCRN and BiFuse in the comparison provided a broader baseline, demonstrating that ViT-Fuse achieved superior performance not only against UniFuse but also against other standard depth estimation frameworks. As presented in Table 2, ViT-Fuse showed significant improvements across multiple metrics. In comparison to UniFuse, ViT-Fuse achieved reductions of 0.0042 and 0.0075 in MAE and RMSE, respectively. The most notable improvement was in the Abs Rel error, where ViT-Fuse outperformed UniFuse by 0.1300, equivalent to approximately 16.46%. On average, ViT-Fuse reduced the overall error metrics by approximately 9.15%, indicating superior predictive performance. Specifically, on the loosest accuracy metric of δ < 1.25^3^, ViT-Fuse showed a modest improvement of 0.34%, while on the tighter metric of δ < 1.25^2^, it achieved a 0.68% increase. The most significant enhancement was observed on the tightest accuracy metric of δ < 1.25, where ViT-Fuse outperformed UniFuse by 0.99%. The enhancement of the accuracy metrics further reinforced the notion that ViT-Fuse achieved greater precision.

**Figure 6:**
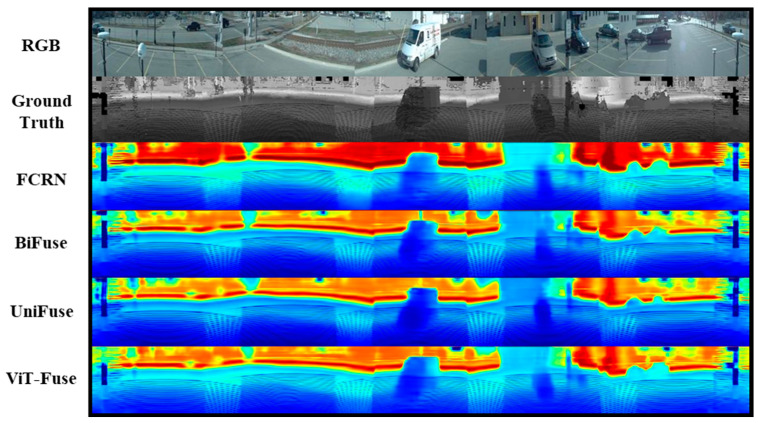
Qualitative results of Ford Campus Vision and LiDAR Dataset.

In comparison to the additional baselines, FCRN and BiFuse, ViT-Fuse also demonstrated superior performance. The qualitative comparison between the FCRN, BiFuse, UniFuse, and ViT-Fuse models is illustrated in Figure 6. ViT-Fuse showed improved depth estimation accuracy and reduced errors, particularly in complex scene structures. The comparison revealed that both FCRN and BiFuse exhibited limitations in capturing detailed object boundaries, leading to less accurate depth maps. In contrast, the combination of the Vision Transformer module and Patch Filling method in ViT-Fuse enabled more precise depth predictions and enhanced the model’s robustness in real-world outdoor environments.

**Figure 7:**
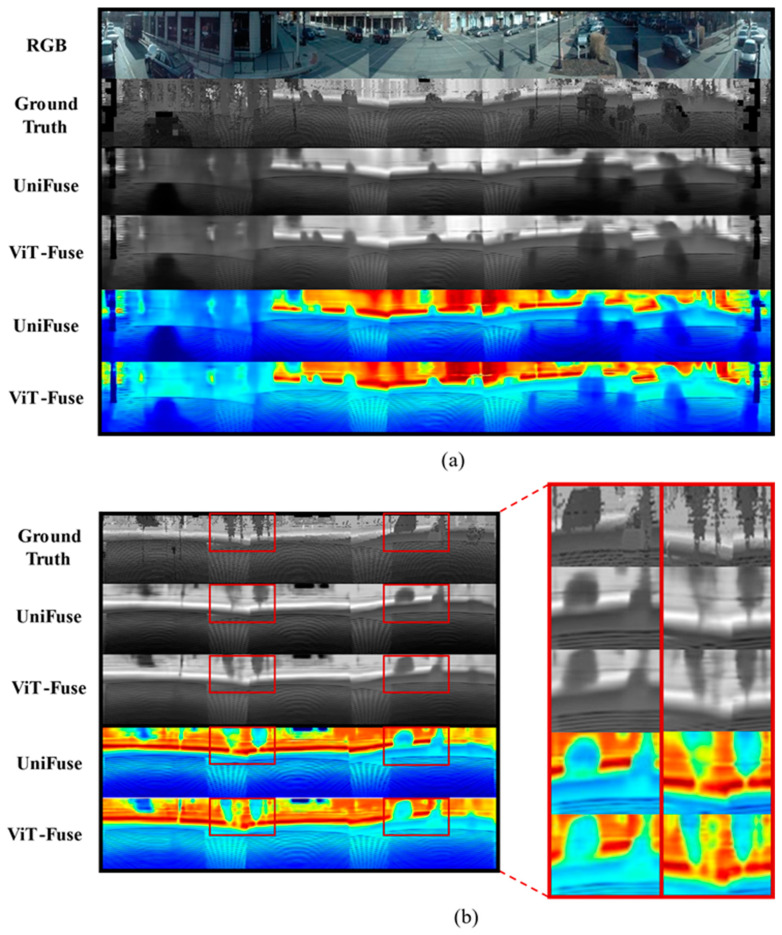
A comparison of the depth prediction effect of the two models is shown in (**a**), and the partial enlarged comparison diagram of the prediction effect of the two models on the edge of the objects is shown in (**b**).

Moreover, since our model was primarily an improvement based on the UniFuse model, we conducted a more detailed comparative validation between the two models on different panoramic images within the dataset. The qualitative comparison between the UniFuse model and our proposed ViT-Fuse model on the Ford Campus Vision and LiDAR Dataset is exhibited in Figure 7. This comparison highlighted the advantages of ViT-Fuse in generating more detailed and accurate depth maps, particularly at the edges of target objects. In Figure 7b, the red-boxed area on the left showed two building groups resembling trees, while the right side contained a vehicle on the road. In the depth map generated by ViT-Fuse, the prediction of the building edges was more precise compared to UniFuse, as observed when comparing the two depth maps side by side. ViT-Fuse exceled in maintaining the integrity of edge information, minimizing the loss that was present in other models’ predictions. Similarly, in the right area, the contours of the vehicle generated by ViT-Fuse were clearer and more comprehensive than those produced by the other models. Both FCRN and BiFuse struggled to capture such fine details, resulting in depth maps that were less accurate along object edges. The ability of ViT-Fuse to accurately represent these contours further demonstrated the effectiveness of the model in mitigating depth prediction errors and optimizing edge performance. These qualitative results, alongside the quantitative improvements in Table 2, further validated the robustness and precision of the proposed ViT-Fuse model.

## 6. Discussion

We designed a panorama depth estimation experiment and tested ViT-Fuse and baseline models on the driving scenario dataset. The ViT module is added to ViT-Fuse, which improves the shortcomings of the traditional convolution with a small receptive field and retains more image information. This is consistent with previous research showing that Vision Transformers can effectively capture global features in image tasks, outperforming CNNs in terms of receptive field size and feature retention [10]. Moreover, the unique self-attention mechanism in the ViT module enhances the ability to aggregate global information, a feature previously explored by Shen et al. [35] in their PanoFormer model to mitigate image distortion in panoramic tasks. Therefore, based on theoretical speculation, ViT-Fuse can optimize the feature fusion process of UniFuse and perform better in panorama depth estimation tasks. The experimental results demonstrate that ViT-Fuse outperforms baseline models, such as UniFuse, with each error metric reduced to varying degrees, aligning with trends observed in prior studies on panoramic image depth estimation [22]. On average, ViT-Fuse reduces overall error metrics by more than 9%, demonstrating a superior performance in comparison to UniFuse, especially in terms of handling complex outdoor scenes and object edges. This further verifies the advantages of using transformers in depth prediction tasks. Although the improvement of ViT-Fuse on the loosest accuracy metric is relatively modest, the model shows remarkable enhancement on the tightest accuracy metric, achieving a 0.99% increase under the δ < 1.25 condition. Such improvements highlight the ability of the ViT-Fuse framework to capture fine details more accurately. Similar advancements in edge-detail handling have been reported in studies involving hybrid CNN–transformer architectures for vision tasks [32].

In addition to the ViT module, the Patch Filling method addresses the sparsity issues in point cloud data by filling gaps with mean interpolation. This complements previous works on depth completion for sparse LiDAR data [11]. The results indicate that the inclusion of Patch Filling not only enhances the model’s accuracy but also improves its robustness in dealing with missing or incomplete observations, which is critical in real-world autonomous driving scenarios.

Despite the promising results achieved by the proposed ViT-Fuse framework, there are several limitations that need to be addressed. First, the experiments were conducted solely on the Ford Campus Vision and LiDAR Dataset, which, while comprehensive, may not fully capture the variety of real-world driving scenarios. This limits the generalizability of our findings across different environmental conditions and datasets. Future works will focus on expanding the scope of validation by conducting experiments on other suitable and available panoramic autonomous driving datasets or on newly created datasets tailored to our research needs. This will provide a more robust understanding of the model’s performance across diverse environments.

Additionally, while the ViT-Fuse model improves feature fusion and depth estimation, the reliance on a single modality—camera-based vision—presents challenges in certain driving conditions, such as poor lighting or adverse weather. In future research, we aim to explore the integration of other sensing modalities, including radar and ultrasonic sensors, to enhance the robustness and accuracy of depth perception. These multi-modal approaches have the potential to improve depth estimation, particularly in situations where visual data alone may be insufficient. Further investigations will also prioritize optimizing the computational efficiency of the ViT module, ensuring the model can be deployed effectively in real-time autonomous driving systems.

## 7. Conclusions

In this paper, the Patch Filling module is designed for the raw 3D point cloud data obtained by the vehicles, which can fill the missing parts of the point cloud appropriately to obtain the true depth information and reduce the prediction error. We validate the Patch Filling method’s effectiveness in solving the sparse point cloud problem by comparing the ViT-Fuse model with and without the Patch Filling. We also have integrated the generated dense depth maps as part of the dataset, improving its usability for future research in panorama depth estimation. In addition, a new panoramic depth estimation model, ViT-Fuse, is proposed for outdoor 360^◦^ autonomous driving images, and it adds the ViT module to depth estimation using ERP and CMP to capture the details of a panoramic image better and enhance the equirectangular projection features. The results show that our model has good performance on the Ford Campus Vision and LiDAR Dataset. The depth map predicted by ViT-Fuse works better on the edge details of the scenes in the map, proving that our method is effective.

## Figures and Tables

**Table 1:** Quantitative results of Patch Filling method on Ford Campus Vision and LiDAR Dataset.

Methods	Error Metric			Accuracy Metric		
	MAE	Abs Rel	RMSE	δ < 1.25	δ < 1.25^2^	δ < 1.25^3^
ViT-Fuse w/o PF	0.09062	0.8188	0.1492	57.34	74.60	82.55
ViT-Fuse w/PF	0.07749	0.6712	0.1314	67.07	82.26	88.78
Improve	−14.49%	−18.03%	−11.93%	+9.73	+7.66	+6.23
ViT-Fuse w/o BI	0.08278	0.7264	0.1376	63.58	79.55	86.91
ViT-Fuse w/o PF	0.07749	0.6712	0.1314	67.07	82.26	88.78
Improve	−6.83%	−8.22%	−4.72%	+3.49	+2.71	+1.87

**Table 2:** Quantitative comparison between our ViT-Fuse model and baseline models.

Methods	Error Metric			Accuracy Metric		
	MAE	Abs Rel	RMSE	δ < 1.25	δ < 1.25^2^	δ < 1.25^3^
FCRN	0.08626	0.8023	0.1515	63.79	80.25	86.99
BiFuse	0.08373	0.7911	0.1450	64.92	81.23	87.98
UniFuse	0.07855	0.7898	0.1333	66.55	82.04	88.76
ViT-Fuse	0.07435	0.6598	0.1258	67.54	82.72	89.10

## Data Availability

Data are contained within the article.
